# Risk Assessment and Pancreatic Cancer: Diagnostic Management and Artificial Intelligence

**DOI:** 10.3390/cancers15020351

**Published:** 2023-01-05

**Authors:** Vincenza Granata, Roberta Fusco, Sergio Venanzio Setola, Roberta Galdiero, Nicola Maggialetti, Lucrezia Silvestro, Mario De Bellis, Elena Di Girolamo, Giulia Grazzini, Giuditta Chiti, Maria Chiara Brunese, Andrea Belli, Renato Patrone, Raffaele Palaia, Antonio Avallone, Antonella Petrillo, Francesco Izzo

**Affiliations:** 1 Division of Radiology, Istituto Nazionale Tumori IRCCS Fondazione Pascale—IRCCS di Napoli, 80131 Naples, Italy; 2 Medical Oncology Division, Igea SpA, 41012 Napoli, Italy; 3 Italian Society of Medical and Interventional Radiology (SIRM), SIRM Foundation, Via della Signora 2, 20122 Milan, Italy; 4 Department of Medical Science, Neuroscience and Sensory Organs (DSMBNOS), University of Bari “Aldo Moro”, 70124 Bari, Italy; 5 Division of Clinical Experimental Oncology Abdomen, Istituto Nazionale Tumori IRCCS Fondazione Pascale—IRCCS di Napoli, 80131 Naples, Italy; 6 Division of Gastroenterology and Digestive Endoscopy, Istituto Nazionale Tumori IRCCS Fondazione Pascale—IRCCS di Napoli, 80131 Naples, Italy; 7 Department of Emergency Radiology, University Hospital Careggi, Largo Brambilla 3, 50134 Florence, Italy; 8 Diagnostic Imaging Section, Department of Medical and Surgical Sciences & Neurosciences, University of Molise, 86100 Campobasso, Italy; 9 Division of Epatobiliary Surgical Oncology, Istituto Nazionale Tumori IRCCS Fondazione Pascale—IRCCS di Napoli, 80131 Naples, Italy

**Keywords:** pancreatic cancer, surveillance, risk assessment, artificial intelligence, radiomics

## Abstract

**Simple Summary:**

Pancreatic cancer (PC) is one of the deadliest cancers. Its high mortality rate is correlated with several explanations; the main one is the late disease stage at which the majority of patients are diagnosed. Since surgical resection has been recognised as the only curative treatment, a PC diagnosis at the initial stage is believed the main tool to improve survival. Therefore, patient stratification according to familial and genetic risk and the creation of screening protocol by using minimally invasive diagnostic tools would be appropriate.

**Abstract:**

Pancreatic cancer (PC) is one of the deadliest cancers, and it is responsible for a number of deaths almost equal to its incidence. The high mortality rate is correlated with several explanations; the main one is the late disease stage at which the majority of patients are diagnosed. Since surgical resection has been recognised as the only curative treatment, a PC diagnosis at the initial stage is believed the main tool to improve survival. Therefore, patient stratification according to familial and genetic risk and the creation of screening protocol by using minimally invasive diagnostic tools would be appropriate. Pancreatic cystic neoplasms (PCNs) are subsets of lesions which deserve special management to avoid overtreatment. The current PC screening programs are based on the annual employment of magnetic resonance imaging with cholangiopancreatography sequences (MR/MRCP) and/or endoscopic ultrasonography (EUS). For patients unfit for MRI, computed tomography (CT) could be proposed, although CT results in lower detection rates, compared to MRI, for small lesions. The actual major limit is the incapacity to detect and characterize the pancreatic intraepithelial neoplasia (PanIN) by EUS and MR/MRCP. The possibility of utilizing artificial intelligence models to evaluate higher-risk patients could favour the diagnosis of these entities, although more data are needed to support the real utility of these applications in the field of screening. For these motives, it would be appropriate to realize screening programs in research settings.

## 1. Background

Cancer represents a leading cause of death and a critical obstacle to increasing life prospects worldwide [1]. According to World Health Organization (WHO), in 2021 [2] cancer was the first or second leading cause of death before 70 years of age in 112 of 183 states, and ranked third or fourth in a further 23 states. Pancreatic cancer (PC) is responsible for a number of deaths (466,000) almost equal to its incidence (496,000) [1], due to its poor prognosis, and represents the seventh leading cause of cancer death in both sexes, with the highest incidence rates in Europe, Northern America, and Australia/New Zealand [1,3]. Considering that incidence and mortality rates have been stable or have slightly increased in many countries, while some cancers such as breast cancer have declined, it is expected that pancreatic cancer will surpass breast cancer as the third leading cause of cancer death by 2025 in a study of 28 European countries [3,4,5,6,7,8,9,10,11,12,13].

The high mortality rate is correlated with several factors, of which the main is the late disease stage at which the majority of patients are diagnosed [14,15,16,17,18,19,20,21,22,23,24,25,26]. In fact, pancreatic cancer is asymptomatic until the disease develops to an advanced stage, and at diagnosis only about 20% of cases are eligible for surgical resection [27,28,29,30,31,32]. In addition, even after the surgical approach, the majority of patients will experience a recurrence, with five-year survival only up to 25% [3,33,34,35,36]. Another critical point is pancreatic cancer biology, which contributes not only to early recurrence and metastasis, but also a resistance to conventional treatment [4,37]. Therefore, in this context, although several alternative treatments have been introduced, as ablative therapies [34,35,36,37,38,39,40,41,42,43,44,45], an effective treatment for this type of tumor remains to be determined [14].

Most primary pancreatic cancers are pancreatic ductal adenocarcinomas (PDACs); only a small proportion are pancreatic neuroendocrine tumors [46,47,48,49,50,51,52,53] and, even rarer, malignancies [54,55,56,57,58,59]. PDACs normally rise through a process that involves several stages, from pancreatic intraepithelial neoplasia (PanIN) [60]. According to pathological sub-type, PanINs can be classified in low (PanIN-1), intermediate (PanIN-2), or high (PanIN-3) grade [60]. Several pieces of research on DNA sequencing have shown a multi-phase process in genetic mutation, with early mutations in KRAS followed by alterations in P1 and P53, among others, with a consequent progression in grade and with PDAC as the final step. [61]. Since PanINs are microscopic, diagnostic tools do not detect these entities, and the diagnosis is correlated with surgical specimen [62,63,64,65,66,67,68]. PanINs surgical resected patients have a five-year survival rate higher than 85% compared to PDAC resected patients [68]. Additionally, a precursor of PDAC could be intraductal papillary mucinous neoplasms (IPMNs) [69,70,71,72,73,74,75,76,77,78,79,80,81]. IPMNs can be classified based on histology and duct involvement (main-duct type or side branch: branch-duct type) [82]. It has been reported, on surgical specimens, that about 20–30% of IPMNs had an invasive tumor, with the highest risk for main-duct sub-types [83]. However, according to several guidelines, not all these lesions need to be resected [80,81,82,83,84,85]. Another entity correlated to PDAC are mucinous cystic neoplasms (MCN), which are slow growing cystic tumors that are normally detected in women [86,87,88]. The risk of progression to malignancy varies between IPMN and MCN, and, considering that the malignant potentials are not fully understood yet, it is critical to identify patients who are at risk in order to supply proper management [89].

## 2. Risk Factors

Although the specific factors correlated to PC onset are not clearly known, several modifiable and non-modifiable features are recognized [90,91,92,93,94,95,96]. Among the non-modifiable risk factors, age, sex, ethnicity, blood group, diabetes mellitus (DM), microbiota, and familial and genetics history and genetic predisposition are recognized. Modifiable risk factors include alcohol, smoking, pancreatitis history, dietary features, and obesity [90,91,92,93].

**Figure 1:**
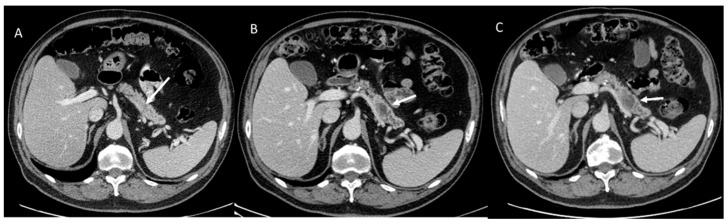
CT evaluation in melanoma patient with FAMM. In (**A**), arrow shows non-detectable lesion. In (**B**) arrow shows PDAC after 1 years (respect to CT assessment of (**A**)) and in (**C**) after 3 months (respect to CT assessment of (**B**)).

**Figure 2:**
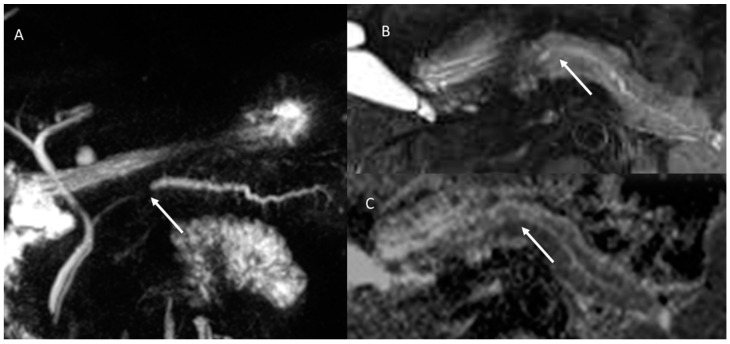
MR/MRCP assessment of Peutz–Jeghers syndrome patient. In MRCP (**A**), the arrow shows black sign and the dilatation of the main and secondary branch ducts. In T2-W sequences the (**B**) arrow shows hyperintense tissue and in ADC map (**C**) hypointense tissue.

**Figure 3:**
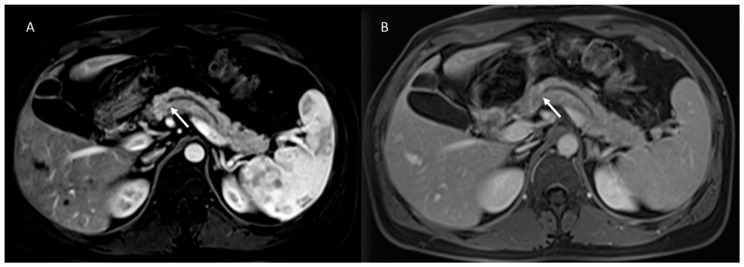
The same patient as in Figure 2; post-contrast MRI evaluation. In arterial (**A**) and portal (**B**) phase of contrast study, arrows show no tissue but dilatation of the main and secondary branch ducts.

With regard to family history and genetic susceptibility, approximately 10% of PC patients have a family PC history, a feature that considerably enhances an individual’s tumor risk [94,95,96,97,98,99,100,101,102,103,104]. Nevertheless, that the genetic profile can influence PC is not well-known; although PC is recognized as a possible lesion in some genetic syndromes, it counts for few familial pancreatic cancer cases [94,95,96,97,98,99,100,101,102,103,104]. With regard to gene mutations, it is known that PC patients can have mutations in BRCA2 while the role of BRCA1 is debatable. In addition, it is known that the mutation in CDKN2A (also known as P16) is correlated with familial atypical mole melanoma syndrome (FAMM) (Figure 1); in STK11 (also known as LKB1), is correlated with Peutz–Jeghers syndrome (Figure 2 and Figure 3); and in PRSS1 and SPINK1, correlated with hereditary pancreatitis. In addition, Lynch syndrome patients, in whom there are mutations in genes encoding DNA mismatch–repair proteins, have a higher risk of PC, as do patient with mutations in PALB2. In addition, another subgroup of familial pancreatic tumor is due to germline mutations in ATM [94,95,96,97,98,99,100,101,102,103,104].

Beyond familial pancreatic cancer, the most well-recognized risk factor for pancreatic tumor is smoking, followed by chronic pancreatitis, diabetes, and obesity, specifically high body-mass index (BMI) and centralized fat distribution [105,106,107,108,109,110,111,112,113,114].

A clear knowledge of these risk factors allows a correct stratification of patients, in the perspective of a surveillance protocol centered on the patient himself [115,116,117,118,119,120,121,122,123,124,125,126,127,128]. However, to avoid the risk of over-diagnosis and to center energy on an early diagnosis, we should define who those subsets of patients are and quantify the degree of risk [115]. Once we have stratified the target population according to risk level, we should define the surveillance timing and the tools (biomarkers and imaging) [115,116,117,118,119,120,121,122,123,124,125,126,127,128]. In the PDAC scenario, we are still interpreting this multi-step process, although definite improvements have been made [115].

## 3. Screening Guidelines 

Several working groups and scientific societies have proposed surveillance protocol for patients at risk of pancreatic cancer [129,130,131,132,133,134,135,136,137,138,139]. All working groups agree that surveillance programs should involve experienced multidisciplinary teams in dedicated oncological settings and that patient starting age should vary according to the underlying genetic condition.

According to the consensus of an international panel of experts, (in 2018, revised in 2019) the International Cancer of the Pancreas Screening (CAPS) Consortium suggested a screening PC program [129]. According to this program, patients with mutations of BRCA2, PALB2, CDKN2A, ATM, or MMR genes* and 1 first-degree relative (FDR) with PC and familial individuals with FPC or two or more BRs and 1 FDR with PC, patients with Peutz–Jeghers or FAMM syndrome, or patients with hereditary pancreatitis after their first attack should be subjected to surveillance [129]. The starting age was 50–55 years for patients with familial risk, 40 for Peutz–Jeghers syndrome or FAMM syndrome, and 45–50 for patients with other mutations. Additionally, for all high-risk patients, surveillance should begin 10 years before the youngest age at which a blood relative developed PC, if this age was lower than the general age guidelines above [129]. 

In 2018, the American Society of Clinical Oncology (ASCO) suggested that familial PC patients should be subjected to risk evaluation [130], proposing that for all patients who developed tumors a germline genetic analysis was useful to evaluate familial predisposition and the need for surveillance. They also suggested that patients with confirmed mutations of APC, ATM, BRCA2, BRCA1, CDKN2A, MMR genes*, PALB2, STK11, or TP53 should be subjected to surveillance [130].

In addition the American College of Gastroenterology (ACG) [140] proposed that patients with germline mutations should be subjected to surveillance. For Peutz–Jeghers syndrome patients, surveillance should start at 35 years (or 10 years younger than earliest PDAC in family); for FAMM syndrome, at 50 years (or 10 years younger than earliest PDAC in family); for Lynch syndrome, at 50 years (or 10 years younger than earliest PDAC in family); for hereditary pancreatitis, at 50 years (or 10 years younger than earliest PDAC in family); for FPC ≥2 relatives with PDAC of whom ≥1 is FDR or ≥3 relatives with PDAC, start at 50 years (or 10 years younger than earliest PDAC in family) [140].

## 4. Screening Modalities

The current PC screening programs are based on the annual employment of magnetic resonance imaging with cholangiopancreatography sequences (MR/MRCP) and/or endoscopic ultrasonography (EUS) [138,141,142,143,144,145,146,147,148,149,150,151,152]. For patients unfit for MRI, computed tomography (CT) could be proposed, although CT shows lower detection rates, compared to MRI, for small lesions [153,154,155,156,157,158,159,160,161,162,163]. According to some of the research, EUS and MR/MRCP could be employed alternatively, or the clinicians should consider patient preference and available expertise [127,128,129,130,131,132,133,134,135,136,137,138,139,140].

Though EUS is now considered the most sensitive imaging modality for the detection of pancreatic lesions, Wiest et al. [164], reported a sensitivity of 88–100%, a specificity of 63.4–94%, a positive predictive value (PPV) of 71.4–96.2%, and a negative (N) PV of 68.5–100% for MRI, that with MRCP has a sensitivity of 97.1–100%, a specificity of 81.8–88%, a PPV of 94.4–97%, and a NPV of 90–100%, similar to EUS, while a lower accuracy was reported for CT. CT sensitivity and specificity are correlated to detector numbers and study protocol [164]. The possibility to add EUS conventional protocol, elastography and/or contrast-enhanced ultrasound (CEUS) could improve the sensitivity of EUS for PC screening [165].

**Figure 4:**
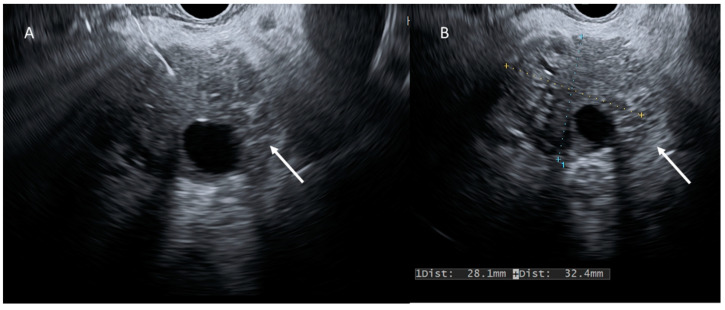
EUS evaluation (**A**,**B**); PDAC (arrows) appears as a heterogeneous hypoechoic mass.

With EUS study, most solid pancreatic lesions are depicted as a heterogeneous hypoechoic mass, irrespective of the pathological type (Figure 4), and this appearance is the same found at transabdominal US [166]. With MRI study, during cholangiopancreatography study, pancreatic lesions appear as a signal blank of the pancreatic duct and in relation to lesion size, they can also be the only sign of disease [167].

However, MRI can allow lesion detection at an earlier stage thanks to the morphological assessment of pancreatic parenchyma, as well as that of the duct. In a meta-analysis, it was reported that MRI and CT had similarly sensitive specifics in PC detection and staging [167]. In addition, the authors compared CT and PET/CT in PC diagnoses, showing inconclusive data, as in the evaluation of CT and EUS-FNA [167]. However, this meta-analysis did not evaluate studies in a screening setting, therefore they were subject to probable bias. 

Conversely, in a study in which the authors evaluated high risk individuals, the overall yield for detecting pre-malignant and tumor lesions using EUS was 20% and using MRI/MRCP was 14% [168]. Notably, EUS had higher performance for solid lesions while MRI performed better for cystic lesions. Therefore, due to higher sensitivities and specificities in small lesions diagnosis, MRI and EUS should be chosen as diagnostic tools in screening programs [167,168,169,170,171,172,173,174]. 

The major limit of EUS and MRI technologies is the inability to reliably detect and distinguish PanINs [94].

## 5. Pancreatic Cystic Neoplasm Diagnostic Management

**Figure 5:**
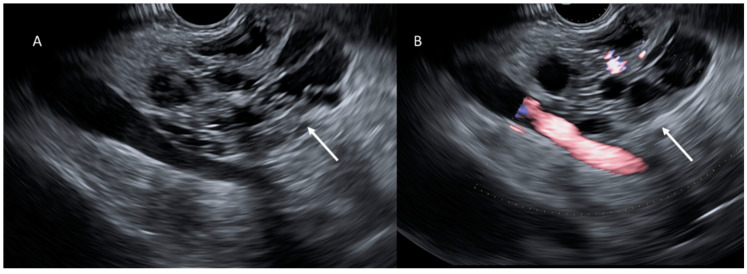
EUS evaluation (**A**,**B**); cystic lesion (arrows) appears as a heterogeneous hypo-isoechoic mass.

**Figure 6:**
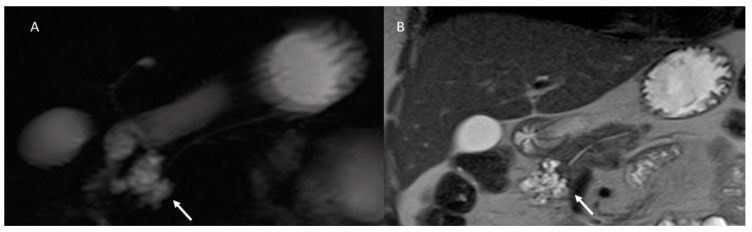
Abbreviated MRI assessment (MRCP: (**A**) and T2-W: (**B**) sequences) of pancreatic cistoadenoma, which is characterized by multicyclic lesions (arrow).

**Figure 7:**
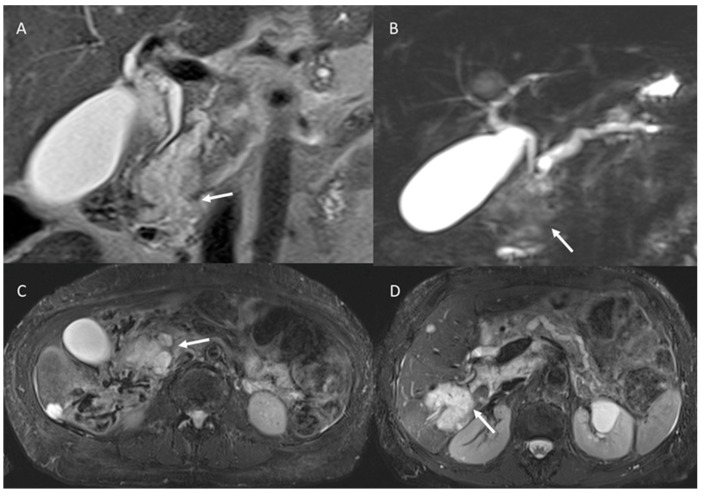
MRI assessment of degenerated MD-IPMN. In T2-W (**A**), MRCP (**B**) and T2-W FS (**C**) sequences the lesions appear (arrows) as a dilated MD with mucinous intraluminal component (arrow). In (**D**) (T2-W FS) arrow shows mucinous liver metastasis.

It is estimated that 2–45% of the general population have pancreatic cystic neoplasms (PCN) [175,176,177,178]. PCNs comprise numerous clinically challenging entities, since their biological behavior varies from benign (Figure 5 and Figure 6) to malignant (Figure 7). In this context, proper management should prevent progression to invasive cancer and should minimize the need for lifelong screening and related costs [179].

Much research has reported an accuracy in recognizing the sub-type of PCN, between 40% and 95% for MRI/MRCP and between 40% and 81% for CT [180,181,182,183,184,185,186,187,188,189], and a CT detection rate of 2.1–2.6% [189,190] and MRI/MRCP detection rate of 13.5–45% [191,192]. However, the diagnostic accuracy is low, using either single or combined imaging tools for differentiating small PCN from non-neoplastic or non-epithelial cysts, or for connection to the ductal system [180,181,182,183,184,185,186,187,188,189,190,191,192].

With regard to diagnostic management, a dedicated pancreatic protocol, either in CT or MRI/MRCP, reported similar accuracy in PCN characterization [193,194,195]. However, MRI/MRCP had a higher accuracy in identifying the communication between a lesion and the duct system, and similarly for septations or the mural nodule [196]. In addition, MRI allows the detection of multiple lesions, which favors a diagnosis of multifocal side-branch IPMN [196,197]. Although MRI is the preferred diagnostic tool, a multimodality assessment should be preferred for tumor staging, or for recurrence diagnosis [198]. 

No specific diagnostic study protocols are recommended for the diagnosis or surveillance of PCN patients, since there is widespread published data and a lack of dedicated comparative studies [198]. 

Several abbreviated protocols (AP) for PC have been proposed [198,199,200,201,202,203,204,205,206,207,208]. For PCN surveillance, AP-MRI could be a good alternative. In fact, several authors suggested MRI protocol without administration of a contrast agent [205]. Macari et al. found that contrast-enhanced images did not lead to different treatment recommendations compared to unenhanced images [205]. Similar results were found by Nougaret et al. [206]. Pedrosa et al. [207] suggested reserving the standard contrast-enhanced MRI protocol with MRCP for the first diagnosis of PCN, while for the follow-up, suggested a 10-min protocol consisting of axial and coronal SSFSE T2-weighted, 2D and 3D single-shot MRCP, and 3D T1-weighted spoiled-gradient echo. With regard to the utility of DWI in the surveillance of pancreatic cystic lesions, there is a debate in the literature [208,209,210,211,212,213]. DWI should be included to avoid the risk of missing a concomitant pancreatic cancer. A combination of T2-W sequences and DWI has been shown to have similar accuracy to a conventional contrast-enhanced MRI protocol [213]. Pozzi-Mucelli et al. showed that that an abbreviated protocol MRI is more economical, and provides equivalent clinical data for patient surveillance [214].

EUS should be considered as an adjunct to other imaging tools, since it allows identifying PCN with features that should be considered for surgical resection, although this tool does not allow us to characterize the PCN sub-type [198]. Contrast-enhanced EUS (CE-EUS) should be considered for the assessment of mural nodules and vascularity within the cyst and septations [198]. In addition, EUS fine-needle aspiration (FNA) of the lesion should be considered for differentiating mucinous versus non-mucinous lesions [198].

With regard to IPMN, jaundice, positive cytology, the presence of a solid component and/or enhancing mural nodule (≥5 mm), or a major pancreatic duct (MPD) measuring ≥10 mm are predictive of malignancy. MPD dilatation between 5 and 9.9 mm, cystic growth-rate ≥5 mm/year, increased level of serum CA 19.9 (>37 U/mL), symptoms, enhancing mural nodules (<5 mm), and/or, for MCN, a cyst diameter ≥40 mm, are also associated with an increased risk for high-grade dysplasia or cancer [138,198].

According to European-based guidelines, IPMN patients which do not meet criteria for surgical resection should be subjected a 6-month follow-up in the first year, and a yearly follow-up thereafter [198].

Regarding mucinous cystic neoplasm (MCN) patients, lesions ≥40 mm should undergo surgical resection [198]. Resection is also suggested for symptomatic patients and for lesions which have risk factors, such as mural nodules [198]. Considering that there has been reported a faster growth during pregnancy, patients with MCN should be observed closely during pregnancy [198]. According to European-based guidelines, MCN <40 mm without mural nodules or symptoms should be subjected to surveillance with MRI, EUS, or a combination of both, every 6 months for the first year, and then annually if no changes are observed [198].

Regarding to the different guidelines, the European guidelines are the most widespread and valid for all PCN patients. The AGA guidelines are designed for asymptomatic patients, but these exclude several very high-risk entities (as MD-IPMN). The ACR guidelines are for all incidental PNCs. The ACG guidelines are designed for any type of PNC, but are the only ones that exclude a possible genetic predisposition. The Fukuoka guidelines are the most specific, since the only target are the IPMN patients [87]. Today, there have not been studies designed to evaluate these guidelines in at-risk patients, consequently, it is not possible to suggest one guideline over another [87].

## 6. Artificial Intelligence, Radiomics, and Pancreatic Cancer

Current technological progresses have allowed the use of artificial intelligence (AI) in medical settings. Since, compared to the human brain, a computer can analyze larger amounts of data, AI could resolve several problems in oncological settings [215,216,217,218,219,220,221,222,223,224]. AI elaborates algorithms, which are qualified to execute meanings that were normally executed by the human brain [225,226,227,228,229,230,231,232,233,234]. Machine learning (ML), a sub-area of AI, uses mathematical models, through the repetition of calculations derived from large amounts of data, and can learn detailed tasks [235,236,237,238,239,240,241,242,243]. These models can be supervised or unsupervised, in relation to the desired outcome of interest in model knowledge [244,245,246,247,248,249]. In their supervised form, a training dataset is introduced to obtain the desired outcome [250,251,252,253,254,255,256,257,258,259,260,261,262,263]. This type of learning requires large amounts of training data which has been pre-labeled (“curated”) by a human operator. Once the training of the model is completed, a different dataset is used to test its performance (testing data) [264,265,266,267,268,269,270,271,272,273,274,275,276,277,278]. In unsupervised learning, the model classifies noncurated data by using the algorithm to identify features within the dataset that can be grouped and analyzed further to reach a specific outcome [279,280,281,282,283,284,285,286,287,288]. 

A new field of interest is radiomics, which analyzes, in a mathematical manner, data obtained by medical images [289,290,291,292,293,294,295,296,297,298,299,300,301,302]. The idea that imaging studies contain a great quantity of data, in the form of grey level patterns, which are imperceptible to the human eye, has become more and more interesting [303,304,305,306,307,308]. These texture features, when correlated with clinical–pathological data and outcomes, theoretically allow diagnostic and prognostic assessment, and could produce evidence-based clinical-decision support systems [309,310,311,312,313,314,315,316,317,318,319]. The main objective is to combine multimodal quantitative data with mathematical methods to provide clear and robust parameters allowing an outcome prediction. Radiomics offers outstanding benefits over qualitative imaging assessment, since this is clearly limited by the subjective evaluation of radiologists. A radiomic information extension can be obtained by adding genomics data (radiogenomics); in fact, genomic markers such as microRNA expression have been shown to be associated with treatment response, metastatic spread, and prognosis that could offer personalized and precision medicine [309,310,311,312,313,314,315,316,317,318,319]. The assessment of textural characteristics, obtained by conventional radiological images, such as CT or MRI, allow the extraction of biological data without an invasive approach while reducing costs and time, avoiding any risk for the patients. For several tumors, radiomic analyses have already provided an accurate evaluation of biology, allowing the identification of features correlated with clinical outcomes [309,310,311,312,313,314,315,316,317,318,319].

In the context of PC, the possibility to identify the lesion at an early stage or in a pre-malignant setting may allow proper management. Therefore, several researches have evaluated AI in PC settings.

Muhammad et al. employed AI to predict the risk of developing PDAC, by assessing demographic data, family history, and comorbidities. Their model was able to predict PC development with good accuracy [320]. Similar data were obtained by Hsieh et al. [321] in patients with type 2 diabetes (T2DM).

Several authors evaluated the accuracy of artificial neural networks (ANNs) in differentiating chronic pancreatitis (CP) from PC on EUS images. Norton et al. [14] evaluated 21 PDAC patients and 14 CP patients, showing that four features had an overall accuracy of 89% [322]. Zhu et al. [323] evaluated 262 PDAC patients and 126 CP patients, reporting that their algorithm reached an overall accuracy of 94%.

With regard to MRI AI and early detection of PC, few studies have been reported. Corral et al. [324] employed a deep learning model to categorize IPMNs. Their algorithm’s sensitivity and specificity for detecting dysplasia were 92% and 52%, respectively [324]; while for detecting high-grade dysplasia or malignancy it had a sensitivity and specificity of 75% and 78%, respectively. 

In an unsupervised algorithm to categorize benign or malignant IPMNs, Hussein et al. [325] obtained an accuracy, sensitivity, and specificity of 58.04%, 58.61%, and 41.67%, respectively.

Two interesting projects are ongoing. The Felix Project, supported by the Lustgarten Foundation and by a team at Johns Hopkins University, is based on deep learning models on 156 PC cases and 300 controls. Preliminary data reported a sensitivity and specificity of 94% and 99%, respectively [326]. The second project, by the Alliance of Pancreatic Cancer Consortium Imaging Working Group, with the scope to create a repository of images, including pre- and post- PC diagnosis of CT, MRI, and US. The end point is to develop AI models that can predict PDAC appearance at an early stage [327].

## 7. Discussion

The main end point of a screening program is reducing PC-related mortality by early-stage tumor diagnosis and/or identifying and treating pre-malignant lesions [328]. Actually, at the time of diagnosis, PC is often metastatic or in a locally advanced stage. Published data from screening programs showed the down-staging of detected PCs, with better survival [329]. Canto et al. assessed 354 high-risk patients with a median follow-up of 5.6 years. Among them, they detected 14 PDACs: 10 (71%) in asymptomatic patients, and 9 early and resectable lesions [329]. Since surgical resection has been recognized as the only curative treatment [149,329,330], a tumor diagnosis in the initial stage is believed to be the main tool to improve survival. Therefore, patient stratification, according to familial and genetic risk, and the creation of screening protocol by using minimally invasive diagnostic tools would be appropriate [198]. 

The potential hazard of screening program comprise adverse events correlated to diagnostic procedures and patient anxiety [132]. Potential over-diagnosis or misdiagnosis could occur, causing an over-treatment of completely benign or low-risk neoplastic lesions [132]. For these motives, it would be appropriate to realize screening programs in the research protocol setting. In fact, the success of these programs requires patient compliance and multidisciplinary team cooperation [132].

The actual major limit is the incapacity to detect and characterize PanIN lesions by EUS and MR/MRCP. The possibility of utilizing AI models to evaluate higher-risk patients could favor the diagnosis of these entities, although more data is needed to support the real utility of these applications in the screening field. 

AI is the primary choice to perform image-based extensive analysis of such minute alterations and identify potential risk predictors for disease. AI systems, as opposed to manual approaches, execute complex tasks without interruption and ensure highly accurate and precise outcomes. In the domain of automated processing and analysis of medical images, AI offers numerous techniques and tools to extract accurate measurements from different structures, and can identify nonlinear features and evaluate tissue properties. For prediction modeling, radiomic analysis and machine and deep learning are regarded as the most reliable and common AI approaches. Recent studies have found that radiomics models contribute greatly to the individualized evaluation of pancreatic lesions, such as tumor detection, classification, differentiation, and antitumor drug-effect prediction [96,331]. In addition, pancreatic cystic lesions have been categorized using radiomics methods in multiple studies [332,333]. Although these findings confirmed the feasibility of radiomics for the assessment of pancreatic cystic lesions [334,335,336,337,338,339,340,341,342,343,344], the robustness of these radiomics diagnostic models may be limited due to the relatively small datasets included in most studies. Therefore, the accumulation of additional research data is required for the study of pancreatic cystic lesions. 

With regard to solid lesions, Qureshi et al. [345] proposed a PDAC risk prediction model using AI analysis of the global features of the pancreas. However, since the morphology of the pancreas was assessed “as a whole”, whether the identified precancerous changes (predictors) were merely the manifestation of local changes that occurred in a specific subregion (presumably where the tumor developed) or all subregions simultaneously adopted such changes remained unknown. To overcome this limit, Javed et al. [346], in a retrospective study, performed an extensive radiomic analysis of the precancerous pancreatic subregions using CT images. The analysis was performed using 324 pancreatic subregions identified in 108 contrast-enhanced abdominal CT scans with equal proportions of healthy control, pre-diagnostic, and diagnostic groups. In a pairwise feature analysis, several textural features were found to be potentially predictive of PDAC. A machine learning classifier was then trained to perform risk prediction of PDAC by automatically classifying the CT scans into healthy control (low-risk) and pre-diagnostic (high-risk) classes, and specifying the subregion(s) likely to develop a tumor. The proposed model was trained on CT scans from multiple phases. Using 42 CT scans from the venous phase, model validation was performed, which resulted in ~89.3% classification accuracy on average, with sensitivity and specificity reaching 86% and 93%, respectively, for predicting the development of PDAC (i.e., high-risk). 

## 8. Conclusions

The main end point of a screening program is reducing PC-related mortality by early-stage tumor diagnosis and/or identifying and treating pre-malignant lesions. Patient stratification, according to familial and genetic risk, and the creation of screening protocol by using minimally invasive diagnostic tools would be appropriate. The actual major diagnostic limit is the incapacity to detect and characterize the PanIN. Artificial intelligence models could evaluate higher risk patients and could favor the diagnosis of these entities.

## Data Availability

Data are reported in the manuscript and at link https://zenodo.org/record/7503163#.Y7VHaHbMK3A.
